# Top-Down Control of Visual Alpha Oscillations: Sources of Control Signals and Their Mechanisms of Action

**DOI:** 10.3389/fnhum.2016.00015

**Published:** 2016-01-20

**Authors:** Chao Wang, Rajasimhan Rajagovindan, Sahng-Min Han, Mingzhou Ding

**Affiliations:** J. Crayton Pruitt Family Department of Biomedical Engineering, University of FloridaGainesville, FL, USA

**Keywords:** granger causality, top-down control, alpha oscillaitons, EEG, working memory, visual spatial attention

## Abstract

Alpha oscillations (8–12 Hz) are thought to inversely correlate with cortical excitability. Goal-oriented modulation of alpha has been studied extensively. In visual spatial attention, alpha over the region of visual cortex corresponding to the attended location decreases, signifying increased excitability to facilitate the processing of impending stimuli. In contrast, in retention of verbal working memory, alpha over visual cortex increases, signifying decreased excitability to gate out stimulus input to protect the information held online from sensory interference. According to the prevailing model, this goal-oriented biasing of sensory cortex is effected by top-down control signals from frontal and parietal cortices. The present study tests and substantiates this hypothesis by (a) identifying the signals that mediate the top-down biasing influence, (b) examining whether the cortical areas issuing these signals are task-specific or task-independent, and (c) establishing the possible mechanism of the biasing action. High-density human EEG data were recorded in two experimental paradigms: a trial-by-trial cued visual spatial attention task and a modified Sternberg working memory task. Applying Granger causality to both sensor-level and source-level data we report the following findings. In covert visual spatial attention, the regions exerting top-down control over visual activity are lateralized to the right hemisphere, with the dipoles located at the right frontal eye field (FEF) and the right inferior frontal gyrus (IFG) being the main sources of top-down influences. During retention of verbal working memory, the regions exerting top-down control over visual activity are lateralized to the left hemisphere, with the dipoles located at the left middle frontal gyrus (MFG) being the main source of top-down influences. In both experiments, top-down influences are mediated by alpha oscillations, and the biasing effect is likely achieved via an inhibition-disinhibition mechanism.

## Introduction

It has been firmly established that posterior alpha oscillations can be modulated in a goal-oriented fashion by attention (Pfurtscheller et al., 1997; Shaw, 2003). When attention is directed to external visual events, alpha power in visual cortex decreases with attention (Worden et al., 2000; Sauseng et al., 2005b; Rajagovindan and Ding, 2011); when attention is directed to internal representations, such as during visual imagery and retention of working memory, alpha power increases with attention (Klimesch et al., 1999; Jensen et al., 2002; Cooper et al., 2003; Tuladhar et al., 2007). Physiologically, the decreased alpha power with external attention is thought to reflect increased excitability over sensory cortices to enhance stimulus processing (Jones et al., 2000; Thut et al., 2006; Romei et al., 2008; Rajagovindan and Ding, 2011; Bauer et al., 2014; Lou et al., 2014), whereas the increased alpha power with internal attention reflects decreased excitability over sensory cortices to gate out sensory input to protect the information maintained in working memory from external interference (Jensen et al., 2002; Klimesch et al., 2007; Jensen and Mazaheri, 2010; Mathewson et al., 2011; Klimesch, 2012).

Goal-oriented sensory biasing is thought to be effected by top-down signals propagating from higher-order brain areas in a topographic and modality specific manner via long-range projections (Kastner and Ungerleider, 2000; Pessoa et al., 2003; van Ede et al., 2010). Traditional approaches to the testing of this hypothesis involve observing changes in sensory cortex by (1) manipulating experimental instructions (Kastner and Ungerleider, 2000; Corbetta and Shulman, 2002; Pessoa et al., 2003; Woldorff et al., 2004), (2) stimulating frontal-parietal networks (Armstrong et al., 2006; Capotosto et al., 2009; Sauseng et al., 2011; Hsu et al., 2014; Jaegle and Ro, 2014), and (3) recording from stroke patients with frontal-parietal lesions (Knight et al., 1999; Barceló et al., 2000; Heilman et al., 2000). Much of the evidence supporting the hypothesis comes from univariate analyses in which neuronal activity from different brain regions is analyzed independently. Stimulation and lesion methods, while powerful, involve the perturbation of the nervous system, and do not yield information on the specific signals that mediate the top-down influence. In the case of naturally occurring lesions such as stroke the extent of damage is not controllable.

Recent work has established the validity of a multivariate statistical method called Granger causality in assessing causal influences in neural systems (Kamiñski et al., 2001; Brovelli et al., 2004; Ding et al., 2006; Bollimunta et al., 2008; Dhamala et al., 2008; Valdes-Sosa et al., 2011; Hu and Liang, 2014). In this study we applied Granger causality to high-density EEG data (128 channels) recorded from human subjects performing two experimental paradigms: (1) a trial-by-trial cued visual spatial attention task (external attention) and (2) a modified Sternberg working memory task (internal attention). Three questions were considered: What are the signals that mediate the top-down regulation of posterior alpha activity? Are these top-down signals issued in a task-specific manner or by a common set of brain areas? How is the sensory biasing achieved by these signals?

## Materials and methods

The experimental protocols were approved by the University of Florida Institutional Review Board. A total of 42 subjects, free from movement and neurological disorders and with normal or corrected-to-normal vision participated in the experiments. All subjects provided and signed written informed consent prior to participation.

### Experimental paradigms

#### Experiment 1: Cued visual spatial attention

**Figure 1:**
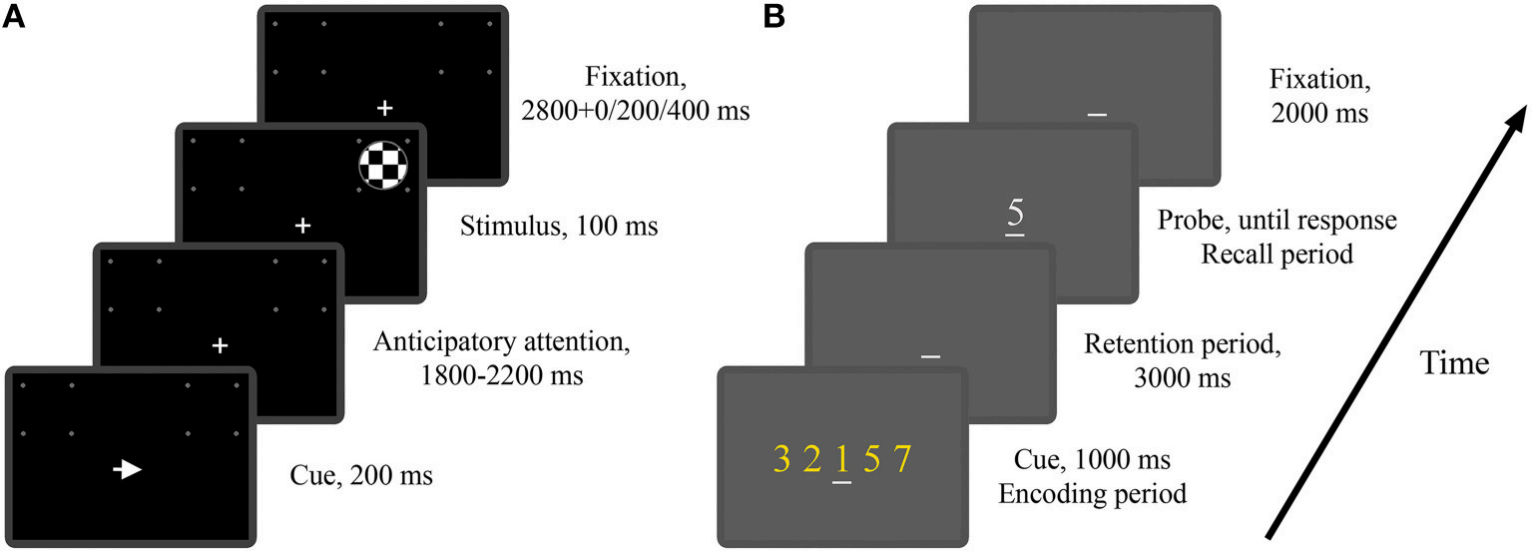
**The two experimental paradigms**. **(A)** Timeline of the visual spatial attention task (Experiment 1). Depicted is a valid trial where the imperative stimulus appeared on the attended side. **(B)** Timeline of the modified Sternberg task (Experiment 2). Depicted is a trial where the memory load is 5 and the probe digit belongs to the cue digit set.

In Experiment 1 twenty one subjects (13 males and 8 females) performed a cued visual spatial attention task. These subjects included the 12 subjects in the study of Rajagovindan and Ding (2011) and 9 additional subjects. As illustrated in Figure 1A, a trial began with the onset of either a left- or right-pointing arrow (200 ms) on a CRT monitor, instructing the subject to deploy covert attention to the square box marked by four white dots in the visual hemifield indicated by the arrow. A “+” sign was used to aid fixation. After a random time delay between 1800 and 2200 ms, a standard or a target stimulus of 100 ms in duration appeared either inside the attended square box (referred to as a valid trial) or inside the square box on the opposite side (referred to as an invalid trial). The standard stimulus was a circular checkerboard subtending a 3.3° visual angle and the target stimulus was also a circular checkerboard whose diameter is 85% that of the standard stimulus. The subject was required to press a button with their right index finger in response to the valid target stimulus as quickly as possible and withhold response to any other stimuli. The standards appeared 80% of the time with 50% validity and the targets appeared 20% of the time with 66% validity. The interval between the cue onset times of two successive trials was randomly varied between 4900 and 5900 ms. The entire experiment comprised 15–16 blocks of trials with 60 trials in each block. Breaks were given between blocks. Subjects received practice sessions of 150 trials to familiarize themselves with the task and to minimize the effect of learning. In this work we are mainly interested in the neural activity during anticipatory attention (Figure 1A).

#### Experiment 2: Modified Sternberg working memory task

In Experiment 2 twenty one subjects (18 males and 3 females) performed a modified version of the classical Sternberg working memory task. As illustrated in Figure 1B, at the beginning of each trial, the subject was shown a set of digits (0–9), referred to as the cue digit set, on a CRT monitor for 1000 ms, which was followed by a 3000 ms interval in which the subject was required to retain the digit set in working memory. At the end of the retention period, a probe digit appeared, and the subject was instructed to press a “yes” (right index finger) or “no” (right middle finger) button to indicate whether the probe digit belonged to the cue digit set. The probe digit would stay on the screen until the subject responded. The interval between the probe offset and the onset of the cue digit set for the next trial was 2000 ms. Memory load was controlled by the size of the digit set which in this experiment was chosen to be 1, 3, or 5. The example trial in Figure 1B has a memory load of 5. A “_” sign was used to aid fixation. The entire experiment consisted of 5 blocks of trials with 60 trials in each block. The three memory loads were equally likely to occur, resulting in 100 trials per memory load. Breaks were given between blocks. Subjects received practice sessions of 100 trials to familiarize themselves with the task and to minimize the effect of learning. By presenting the digits all at once rather than sequentially as in the classical Sternberg task (Sternberg, 1966), the periods of encoding, retention and recall are well delineated temporally, allowing us to study both the temporal and spatial development of neural activity during the different stages of working memory process (Jensen et al., 2002). In this work we are mainly interested in the neural activity during working memory retention (Figure 1B).

### Data acquisition

Scalp EEG data was recorded inside an acoustically and electrically shielded room with a 128-channel BioSemi Active Two System at a sampling rate of 1024 Hz. Four additional electrodes were placed around the eyes to measure electrooculogram (EOG). Stimuli were delivered via the BeriSoft Experimental Run-Time System (ERTS) and key press responses were registered by an EXKEY microprocessor logic pad (http://www.berisoft.com).

### Data preprocessing

Data preprocessing was performed off-line using BESA 5.3 (www.besa.de), EEGLAB (http://sccn.ucsd.edu/eeglab/), and custom scripts written in MATLAB (www.mathworks.com). The original continuous data were high-pass filtered at 0.5 Hz and low-pass filtered at 83 Hz. Both the high-pass and low-pass filters are zero-phase FIR filters which filter the data both forward and backward to ensure that phase delays introduced by each filter are nullified. After filtering, data were downsampled to 250 Hz, and re-referenced against the average reference. For the spatial attention task, the data from the anticipatory attention period, −500 to 0 ms, with 0 ms denoting stimulus onset was selected for further analysis. This time period was chosen for two reasons. First, by choosing a time period immediately preceding stimulus onset, we were able to more effectively examine how anticipatory attention biases visual activity to facilitate stimulus processing. Second, prior studies (Worden et al., 2000; Thut et al., 2006) have shown a sustained attentional modulation of alpha activity throughout this period. For the working memory task, the data from the retention period, −2000 to −1000 ms, with 0 ms denoting probe onset was selected for further analysis. This time period was chosen according to a time-frequency analysis which found that visual alpha activity in this time period was most significantly modulated by memory load. For the time period immediately preceding probe onset, alpha activity declined, possibly due to increased anticipation of probe processing.

To match the 500 ms length of the analysis window in the spatial attention task, the selected 1000 ms retention period in the working memory task was separated into two non-overlapping 500 ms windows for analysis. Results from the two non-overlapping windows were then combined by averaging. For both experiments, artifacts including eye movements and eye blinks, temporal muscle activity and line noise were removed from data epochs using the Infomax ICA algorithm implemented in EEGLAB according to established procedures (Jung et al., 2000). It has been pointed out that ICA-based artifacts rejection is compatible with Granger causality analysis (Seth, 2010). Trials with incorrect responses and with residual artifacts (activity exceeding 75 μV in any of the 128 scalp channels) were excluded from further analysis. For the spatial attention task, ~13% of the trials were rejected due to behavioral reasons and ~11% of the trials were rejected due to artifacts. For the working memory task, ~1% of the trials were rejected due to behavioral reasons and ~20% of the trials were rejected due to artifacts. The longer time period of the working memory task increases the likelihood of data contamination and is likely the reason for the higher artifacts rejection rate.

### Data analysis

#### Sensor level data

To standardize the electrode positions across subjects, the 128 channel data were mapped onto the standard 81 channel (10-10 system) montage using the BESA software (Perrin et al., 1989; Nunez et al., 1997; Scherg et al., 2002). The scalp current source density (CSD) estimates were then computed from the surface potentials to mitigate the adverse impact of volume conduction and common reference on connectivity analysis (Srinivasan et al., 2007).

#### Source level data

**Figure 2:**
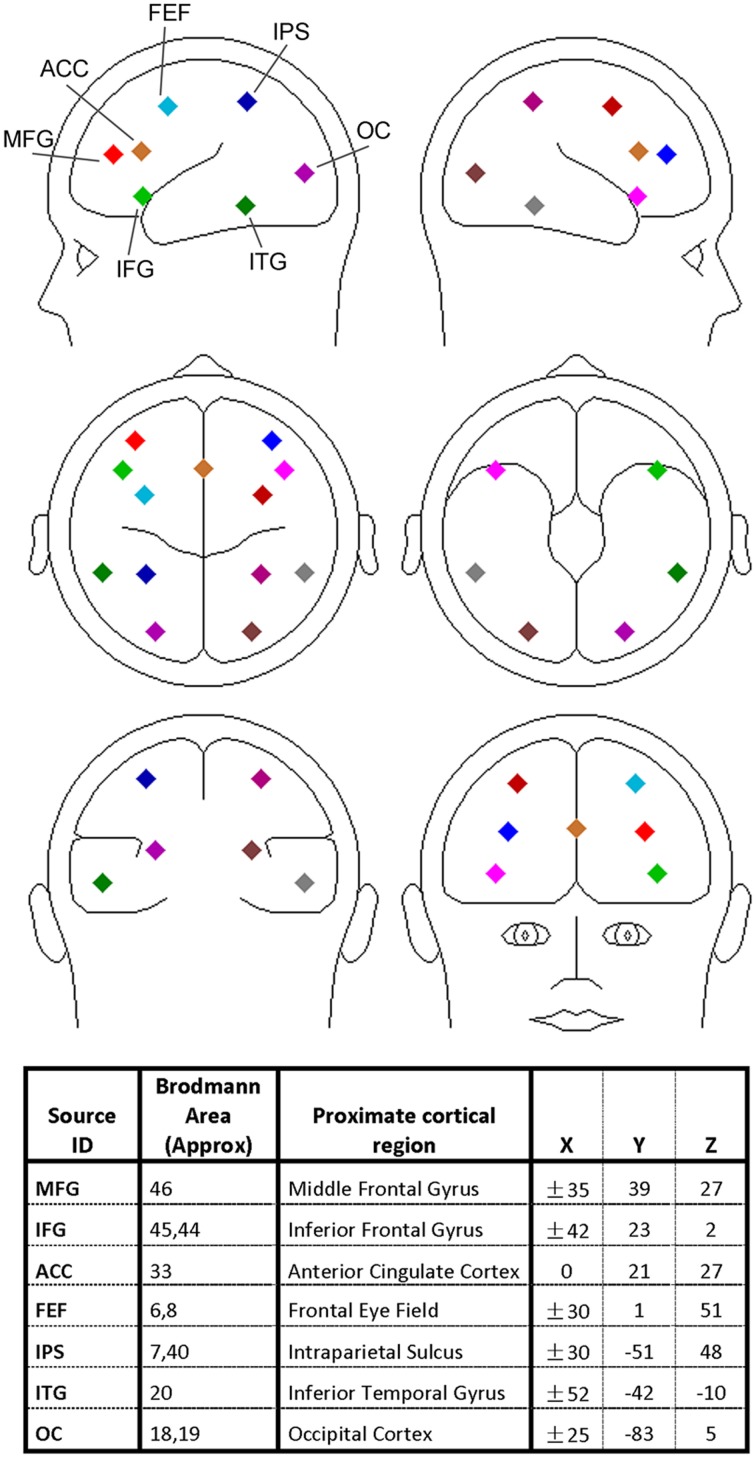
**Regional dipole sources used to convert sensor-level data to source-level data**. The Talairach coordinate system was used.

The discrete dipole source modeling technique implemented in the BESA software (Scherg, 1992) was applied to map the activities of the different brain regions of interest in the source space (Hoechstetter et al., 2004; Keil et al., 2009; Silton et al., 2010; Anderson and Ding, 2011; Adhikari et al., 2014). In this technique a four-shell ellipsoidal head model (Berg and Scherg, 1994) was used and the inverse solution was obtained by a least-squares algorithm. Taking into account of both spatial coverage and the specificity of the brain regions known to be associated with visual spatial attention and verbal working memory, we seeded 13 symmetric regional sources into the brain regions commonly activated in both types of experiments, including bilateral middle frontal gyrus (MFG), bilateral inferior frontal gyrus (IFG), anterior cingulate cortex (ACC), bilateral frontal eye field (FEF), bilateral intraparietal sulcus (IPS), bilateral inferior temporal gyrus (ITG), and bilateral occipital cortex (OC); see Figure 2 for graphical representations and Talairach coordinates of the 13 sources (BESA uses the Talairach coordinate system). Each regional source is composed of three spatially orthogonal current dipoles with the orientation of one dipole set to be radial to the head surface. The same set of regional sources was used for both experiments for two reasons: (1) the brain regions critically involved in spatial attention and working memory exhibit a remarkable degree of overlap (LaBar et al., 1999; Pessoa and Ungerleider, 2004) and (2) use of the same set of brain regions facilitates comparison between the two experiments. The coordinates of these sources were derived from previous neuroimaging studies of visual spatial attention (Corbetta et al., 1993, 1998; LaBar et al., 1999; Hopfinger et al., 2000) and verbal working memory (Jonides et al., 1997; LaBar et al., 1999; Gruber and von Cramon, 2003; Veltman et al., 2003; Walter et al., 2003; Crottaz-Herbette et al., 2004; Koppelstaetter et al., 2008; Michels et al., 2010). In particular, because visual activation is often not reported in verbal working memory studies, the occipital sources used in Experiment 1 was used in Experiment 2. In Supplementary Figure 6 we verified that EEG alpha activity localized to these occipital sources was modulated by working memory load.

A source sensitivity map constructed with the BESA software (Scherg et al., 2002) suggests that each regional source mainly accounts for local neural activity (see Supplementary Figure 1). It is worth noting that for a small brain region like FEF the corresponding source is likely to pick up activity beyond the region. Furthermore, the locations of the sources should be considered approximate as no structural images were obtained from the participants. However, past work has found that the source waveforms from multiple-dipole modeling, in which a regional dipole source is used to model several gray matter patches in its vicinity, are relatively insensitive to small changes in source locations (Scherg et al., 2002; Anderson and Ding, 2011).

#### Multivariate autoregressive modeling

Scalp CSD data and regional dipole data were treated as time series and subjected to multivariate autoregressive (MVAR) modeling from which power and Granger causality spectra were derived (Ding et al., 2000, 2006). For each experimental condition (attend-left or attend-right for Experiment 1; memory load 1, 3, or 5 for Experiment 2), the ensemble mean, which is the average over trials triggered on the cue onset, in the analysis window was estimated and subtracted from single trial time series to ensure that the residuals can be treated as coming from a zero-mean stochastic process, a step required by MVAR data modeling. Model estimation was done separately for scalp CSD data and regional dipole data. A model order of 20 (80 ms in time for a sampling rate of 250 Hz) was chosen as determined by comparing the spectral estimates obtained by the MVAR model and that by the Fourier based method for data pooled across all subjects (see Supplementary Figure 2). Although MVAR model based spectral analysis has infinite frequency resolution, for calculating power and Granger causality in a given frequency band, the frequency step was set to be 0.2 Hz.

#### Spectral power analyses

Power spectral densities (PSDs), derived from MVAR modeling, were estimated at the scalp CSD level for each channel in each subject. To facilitate averaging across subjects the estimated power spectra were normalized within each subject before averaging. Specifically, for the spatial attention experiment, the estimated power spectra for attend-left and attend-right conditions were normalized by the mean alpha power (8–12 Hz) of the attend-right condition. For the working memory experiment, the estimated power spectra for each memory load were normalized by the mean alpha power (8–12 Hz) of the load-1 condition. A Wilcoxon signed-rank test (Wilcoxon, 1945) was employed to test whether the difference between two experimental conditions in a given frequency band was statistically significant.

#### Granger causality analysis

**Figure 3:**
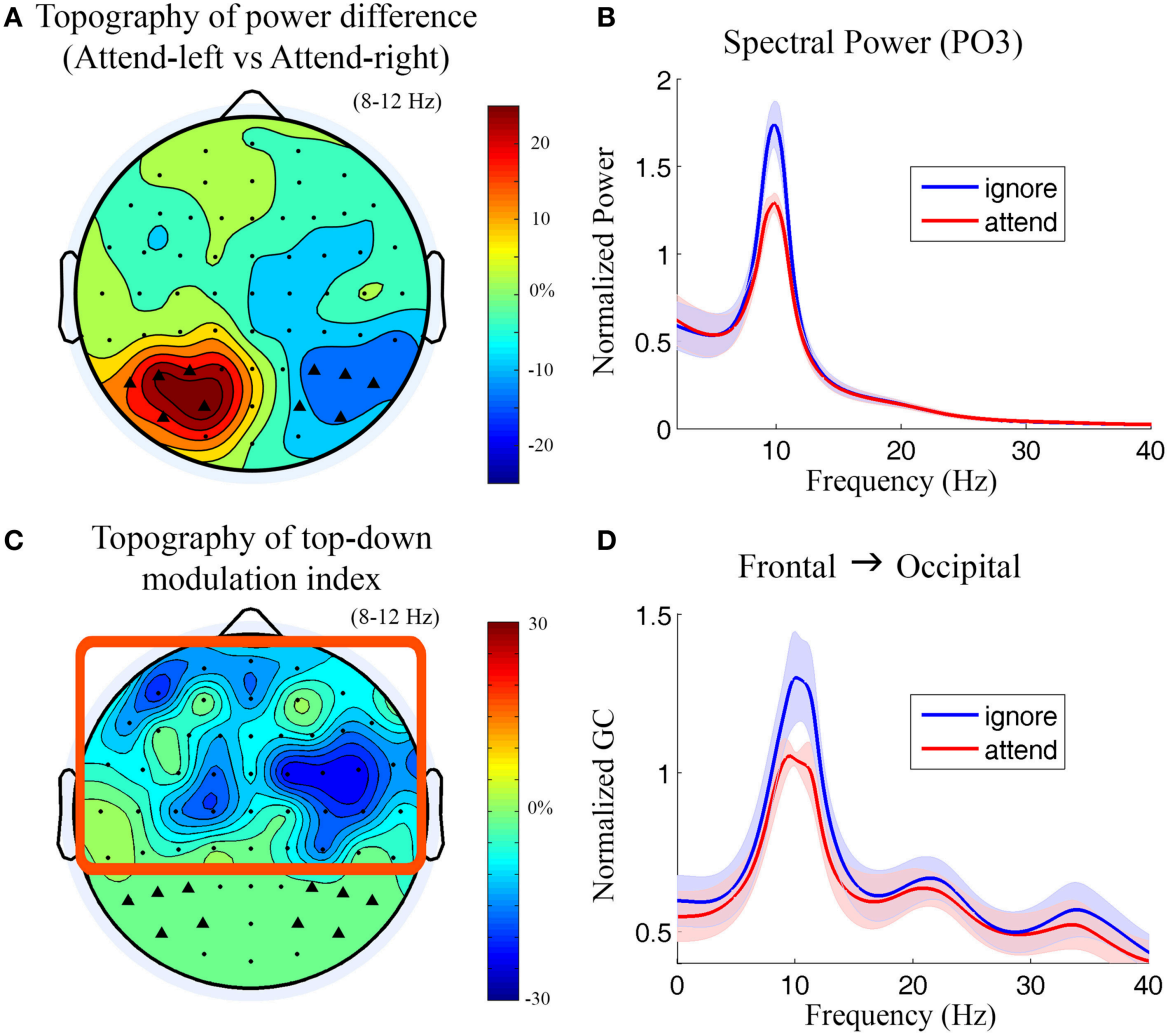
**Power and Granger causality analysis at the scalp CSD level for Experiment 1**. **(A)** Topographical map of percentage change of alpha power by contrasting attend-left condition against attend-right condition. The channels which showed strong alpha modulation were marked by black triangles and selected as sensory channels of interest for Granger causality analysis. **(B)** Grand average power spectra from a left posterior channel (PO3) under attend (attend-right) and ignore (attend-left) conditions. The shaded area indicates the standard error of the mean. **(C)** Topographical map of top-down modulation index (TDMI) in the alpha band. Channels with high TDMI values are channels whose causal influences to the marked occipital channels are highly modulated by spatial attention. Channels inside the square box are considered top channels. Here the analysis window is −500 to 0 ms with 0 ms denoting stimulus onset. **(D)** Grand average Granger causality spectra from a right frontal channel (FC4) to the marked occipital channels (black triangles) under attend and ignore conditions (see Methods).

Frequency-domain Granger causality (GC) analysis (Geweke, 1982; Ding et al., 2006) was performed both at the scalp CSD level and at the source level. At the scalp CSD level, the occipital channels that showed strong alpha power modulation by task conditions (attend-left vs. attend-right in Experiment 1 and load-5 vs. load-1 in Experiment 2) were selected in each experiment based on the inspection of the topographic maps in Figures 3A, **5A**, and marked by black triangles. For each experiment the same occipital channels were used for all participants; different occipital channels were used for different experiments. The channels in the frontal, parietal, and temporal scalp regions (shown inside the square box in Figures 3C, **5C**) were considered top-channels, and GC from each top-channel to occipital channels was calculated. To represent task specific top-down modulation, a top-down modulation index (TDMI) was computed by the following procedure. Let the Granger causality at frequency *f* from a top channel *i* to a visual cortex channel *j* for Subject *k* be denoted as *GC*_*i*→*j*_(*k, f*). For the spatial attention experiment, the selected visual channels were separated into left-hemisphere and right-hemisphere groups. For a left visual cortex channel *l*, attend-left is the ignore condition and attend-right is the attend condition. For a right visual cortex channel *r*, attend-left is the attend condition and attend-right is the ignore condition. Combining results across hemispheres, the causal influence (*CI*) from a top channel *i* on visual cortex under the attend condition was calculated as

$$\begin{array}{l} CI_{i}\left(k,f\right){|}_{attend}=[\frac{1}{L}\sum_{l=1}^{L}GC_{i\to l}\left(k,f\right){|}_{attend\;right}\\ \;+\frac{1}{R}\sum_{r=1}^{R}GC_{i\to r}\left(k,f\right){|}_{attend\;left}]/2 \end{array}$$

where *L* and *R* are the total numbers of the selected visual cortex channels in left and right hemispheres, respectively. Similarly, the causal influence from a top channel *i* on visual cortex under the ignore condition was calculated as

$$\begin{array}{l} CI_{i}\left(k,f\right){|}_{ignore}=[\frac{1}{L}\sum_{l=1}^{L}GC_{i\to l}\left(k,f\right){|}_{attend\;left}\\ \;+\frac{1}{R}\sum_{r=1}^{R}GC_{i\to r}\left(k,f\right){|}_{attend\;right}]/2 \end{array}$$

The TDMI of channel *i* was then defined as the percent change of its causal influence on visual cortex between the attend condition and the ignore condition relative to the attend condition, namely,

$$\begin{array}{l} TDMI_{i}\left(k,f\right)\;=\;[CI_{i}\left(k,f\right){|}_{attend}\\ \;-\;CI_{i}\left(k,f\right){|}_{ignore}]/CI_{i}{\left(k,f\right)}_{attend} \end{array}$$

For the working memory experiment, the causal influence from a top channel *i* on visual cortex under memory load *x* was calculated as $CI_{i}\left(k,f\right){|}_{loadx}=\frac{1}{M}\sum_{j=1}^{M}\;GC_{i\to j}(k,f){|}_{loadx}$, where *M* is the total number of selected visual cortex channels. The TDMI of channel *i* was then defined as the percent change of its causal influence on visual cortex between memory load-5 and memory load-1 relative to memory load-1, namely,

$$TDMI_{i}\left(k,f\right)=[CI_{i}\left(k,f\right){|}_{load5}-CI_{i}\left(k,f\right){|}_{load1}]/CI_{i}{\left(k,f\right)}_{load1}$$

The TDMIs calculated above were then averaged across subjects for each top channel for each frequency band.

At the source level, top-down Granger causality spectra from each of the frontal, parietal and temporal sources (top sources) to the bilateral occipital sources were computed. The TDMI of each source was calculated in a similar way as we described for the scalp CSD level data. Because each regional source is composed of three spatially orthogonal dipoles, for each experimental condition, the magnitude of the Granger causality spectrum for each regional source pair was computed by taking the square root of the sum of the squares of each dipole pair's Granger causality spectrum (9 pairs). The reason we considered all three orthogonal dipoles in our computation is because (1) different gray matter patches in the source's vicinity may contribute differently to each of the three dipoles and (2) using only one dipole may under-represent the contribution of some gray matter patches.

The Wilcoxon signed-rank test (Wilcoxon, 1945) was used to test the significance of the TDMI measures at both the scalp CSD level and at the source level.

## Results

### Experiment 1: The visual spatial attention task

#### Behavioral analysis

The data from two participants were excluded due to poor performance (target detection rates less than 65%). For the remaining 19 subjects (20–31 years of age, 8 females, 17 right-handed) the average target detection rate was 87.2% (*SD* = 11.6%) and the mean reaction time to the attended target was 719 ms (*SD* = 143 ms). Target laterality had no significant effect on target detection rate and reaction time (*p* > 0.05). The average false alarm rates stemming from (1) responding to the target appearing in the ignored location, (2) responding to the attended standard stimulus, and (3) responding to the ignored standard stimulus were 2.9% (*SD* = 2.3%), 6.0% (*SD* = 4.6%), and 1.9% (*SD* = 0.8%), respectively.

#### Sensor level analysis

During anticipation of the impending stimulus (−500 to 0 ms with 0 ms denoting stimulus onset), the topography of alpha power difference between attend-left and attend-right conditions is shown in Figure 3A. It can be seen that alpha activity was maximally suppressed over the visual areas contralateral to the attended visual field (see Supplementary Figure 4 for t-maps). The power spectra from a left posterior channel (PO3) are plotted in Figure 3B. The power of alpha activity (8–12 Hz) for attend condition (attend-right) was significantly lower (*p* = 0.00035) than that for ignore condition (attend-left). The channels which showed strong alpha modulation in Figure 3A were marked by black triangles and selected as sensory channels of interest for the subsequent Granger causality analysis.

Figure 3C shows the topography of top-down modulation index (TDMI) in the alpha range. Channels with higher magnitudes of TDMI indicate that their causal influences onto occipital channels are more modulated by spatial attention. It can be seen that the most prominent effect appears to be localized to channels lying over the right frontal cortex. Granger causality spectra from a right frontal channel (FC4) to the marked occipital channels under attend and ignore conditions are plotted in Figure 3D. A significant decrease in alpha (8–12 Hz) Granger causality was seen in attend condition when compared with ignore condition (*p* = 0.024). Granger causality values in theta (4–7 Hz), beta (15–30 Hz), and low gamma bands (30–40 Hz) were not significantly modulated by attention (*p* > 0.05).

#### Source level analysis

**Figure 4:**
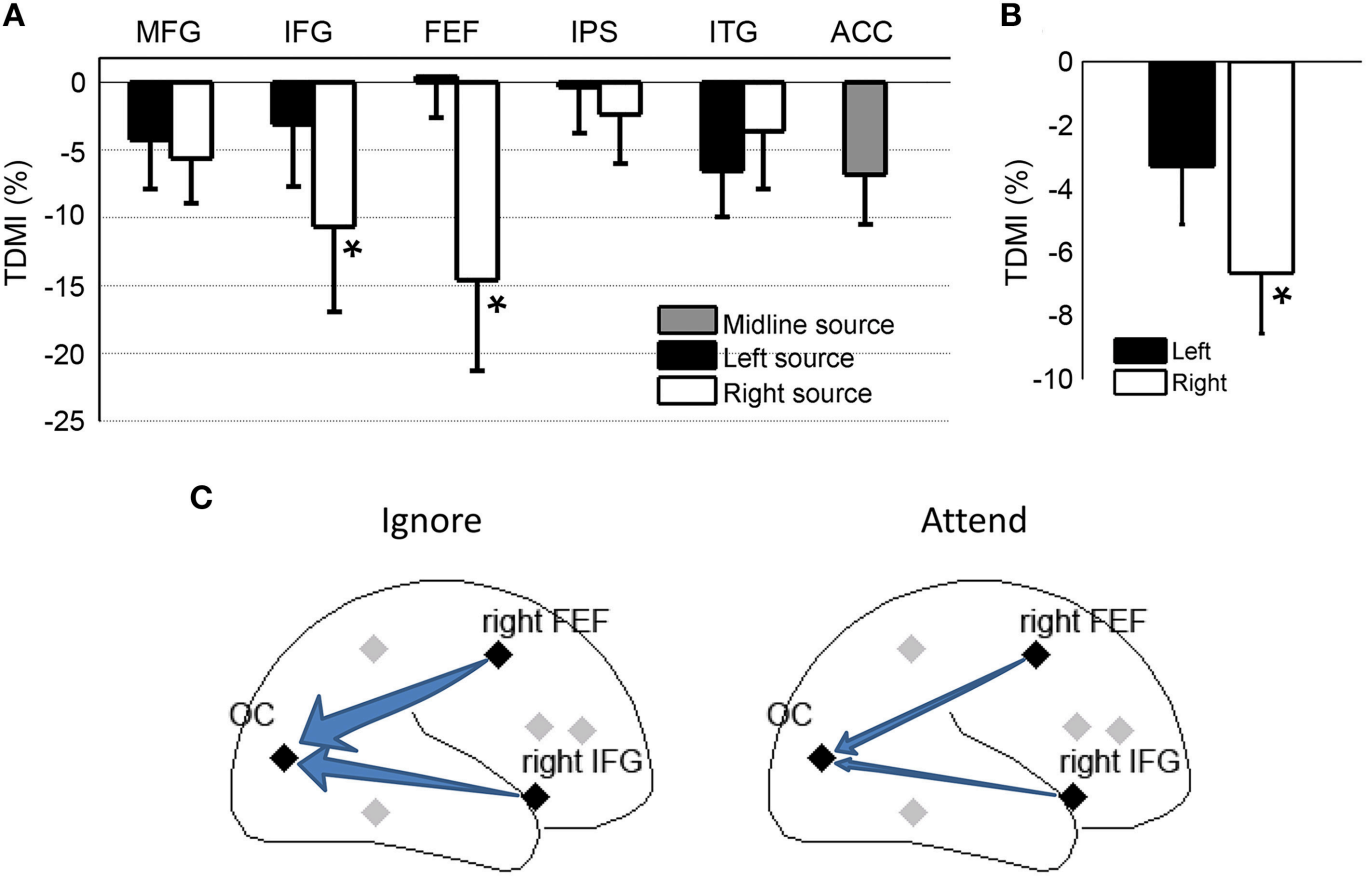
**Granger causality analysis at the source level for Experiment 1**. **(A)** Top-down modulation index (TDMI) of regional sources during anticipatory visual spatial attention. MFG, middle frontal gyrus; IFG, inferior frontal gyrus; FEF, frontal eye field; IPS, intraparietal sulcus; ITG, inferior temporal gyrus; ACC, anterior cingulate cortex. **(B)** TDMI of left hemisphere sources and right hemisphere sources ^*^p < 0.05. **(C)** Schematic showing that alpha-band causal influences, the right FEF→OC (occipital cortex) and the right IFG→OC, significantly decreased in the attend condition compared to the ignore condition.

For most of the frontal, parietal and temporal regional sources, the TDMI values in the alpha frequency range are negative, indicating that the causal influences from these sources onto the occipital sources were lower in the attend condition compared to the ignore condition (Figure 4A). Statistically, the causal influences for right FEF→OC and right IFG→OC were significantly decreased in the attend condition than the ignore condition (*p* = 0.039 for right FEF→OC, *p* = 0.048 for right IFG→OC), suggesting that right FEF and right IFG are likely the major sources of top-down signals for biasing visual cortical activity in covert visual spatial attention (Figure 4A).

Averaging the sources by hemisphere (Figure 4B), we found that the mean index from the right hemisphere sources was significantly less than zero (*p* = 0.0019), whereas the mean index from the left hemisphere sources was not significantly different from zero (*p* > 0.05). The difference between left and right hemispheres, however, did not reach significance (*p* > 0.05). Figure 4C schematically shows that the alpha-band causal influences for right FEF→OC and right IFG→OC significantly decreased in the attend condition when compared to the ignore condition.

### Experiment 2: The modified sternberg working memory task

#### Behavioral analysis

All 21 subjects performed the task according to instructions. Data from 18 subjects (18–35 years of age, 3 females, all right-handed) were included in the analyses. Three participants were excluded due to excessive EEG artifacts. The mean reaction time averaged across subjects was 585 ms (*SD* = 161 ms) for load-1, 763 ms (*SD* = 222 ms) for load-3, and 848 ms (*SD* = 210 ms) for load-5. The mean error rate averaged across subjects was 0.9% (*SD* = 1.0 %) for load-1, 1.1% (*SD* = 0.9%) for load-3, and 1.1% (*SD* = 1.1%) for load-5.

#### Sensor level analysis

**Figure 5:**
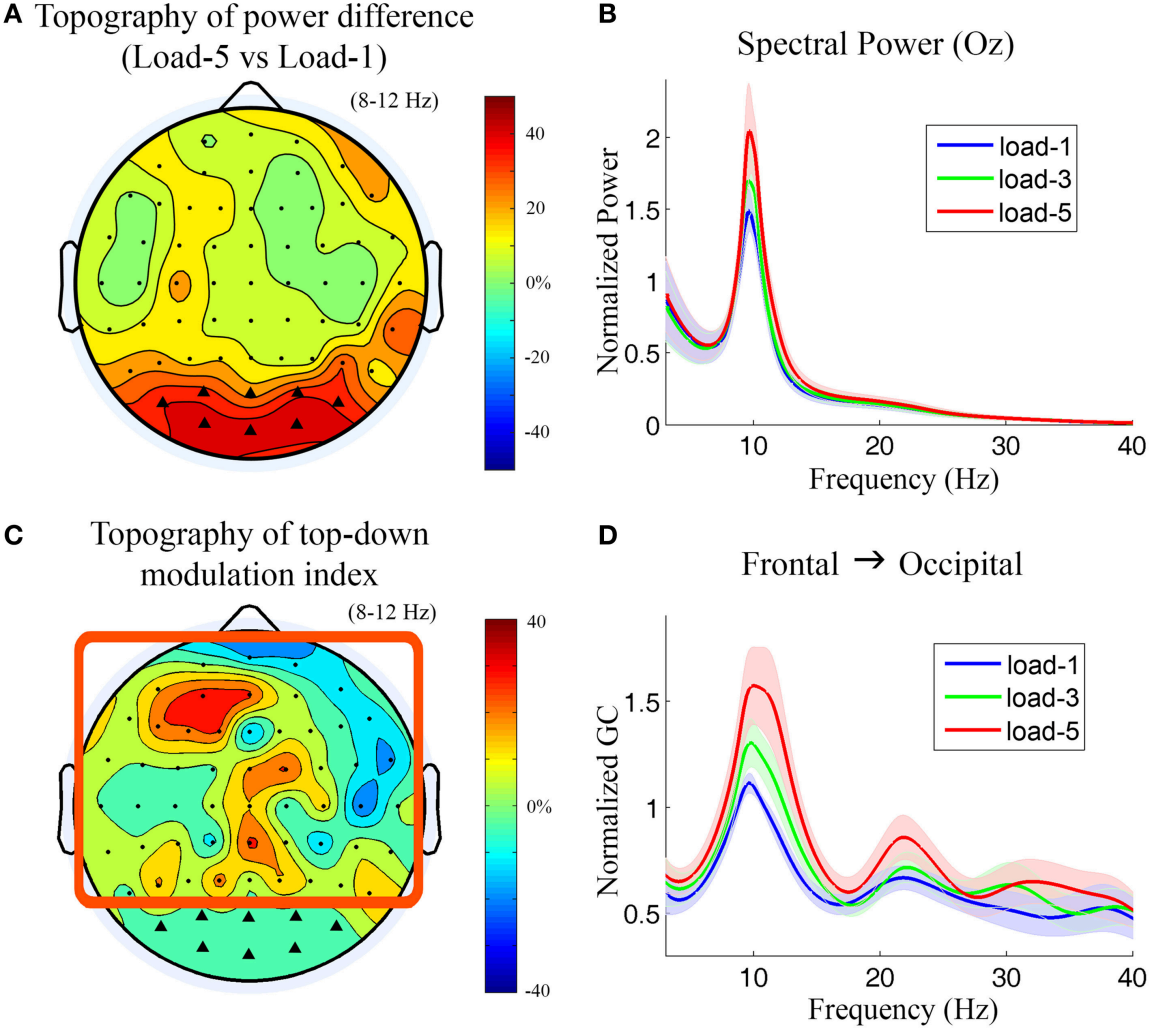
**Power and Granger causality analysis at the scalp CSD level for Experiment 2**. **(A)** Topographical map of the alpha power percentage change between load-5 and load-1. Channels showing strong alpha modulation were marked by black triangles and selected as sensory channels of interest for Granger causality analysis. **(B)** Grand average power spectra from an occipital channel (Oz) under load-1, load-3, and load-5 conditions. The shaded area indicates the standard error of the mean. **(C)** Topographical map of the top-down modulation index (TDMI) in the alpha band. The channels with high TDMI values mean that their causal influences to the marked occipital channels are highly modulated by working memory load. Channels inside the square box are considered top channels. Here the analysis window is −2000 to −1000 ms with 0 ms denoting probe onset. **(D)** Grand average Granger causality spectra from a left frontal channel (AF3) to the marked occipital channels under load-1, load-3, and load-5 conditions.

During working memory retention (−2000 to −1000 ms with 0 ms denoting probe onset), the topographical map of the alpha power differences between load-5 and load-1 (Figure 5A) shows that the most prominent alpha power increases under the high memory load condition are over the bilateral and central occipital regions. The power spectra from a central occipital channel (Oz) are shown in Figure 5B for different memory loads. Alpha power was significantly increased with working memory load (load-5 > load-1, *p* = 0.0026; load-5 > load-3, *p* = 0.0014; load-3 > load-1, *p* = 0.010). The power in theta, beta and gamma frequency range was not significantly modulated by memory load (*p* > 0.05). The channels which showed strong memory load modulation in Figure 5A were marked by black triangles and selected as sensory channels of interest for the subsequent Granger causality analysis.

Figure 5C shows the topography of TDMIs in the alpha range. The most prominent effect appears to be localized to channels lying over the left prefrontal cortex. This is in contrast to what we found in Experiment 1 where the right frontal cortex showed the most prominent effect (Figure 3). The Granger causality spectra from a left frontal channel (AF3) to the marked occipital channels are plotted in Figure 5D. It can be seen that the alpha-band Granger causality increased with working memory load (load-5 > load-1, *p* = 0.017; load-3 > load-1, *p* = 0.040; load-5 > load-3, *p* = 0.18) although the increase from load-3 to load-5 is not significant. The Granger causality values in theta, beta and low gamma bands were not significantly modulated by memory load (*p* > 0.05).

#### Source level analysis

**Figure 6:**
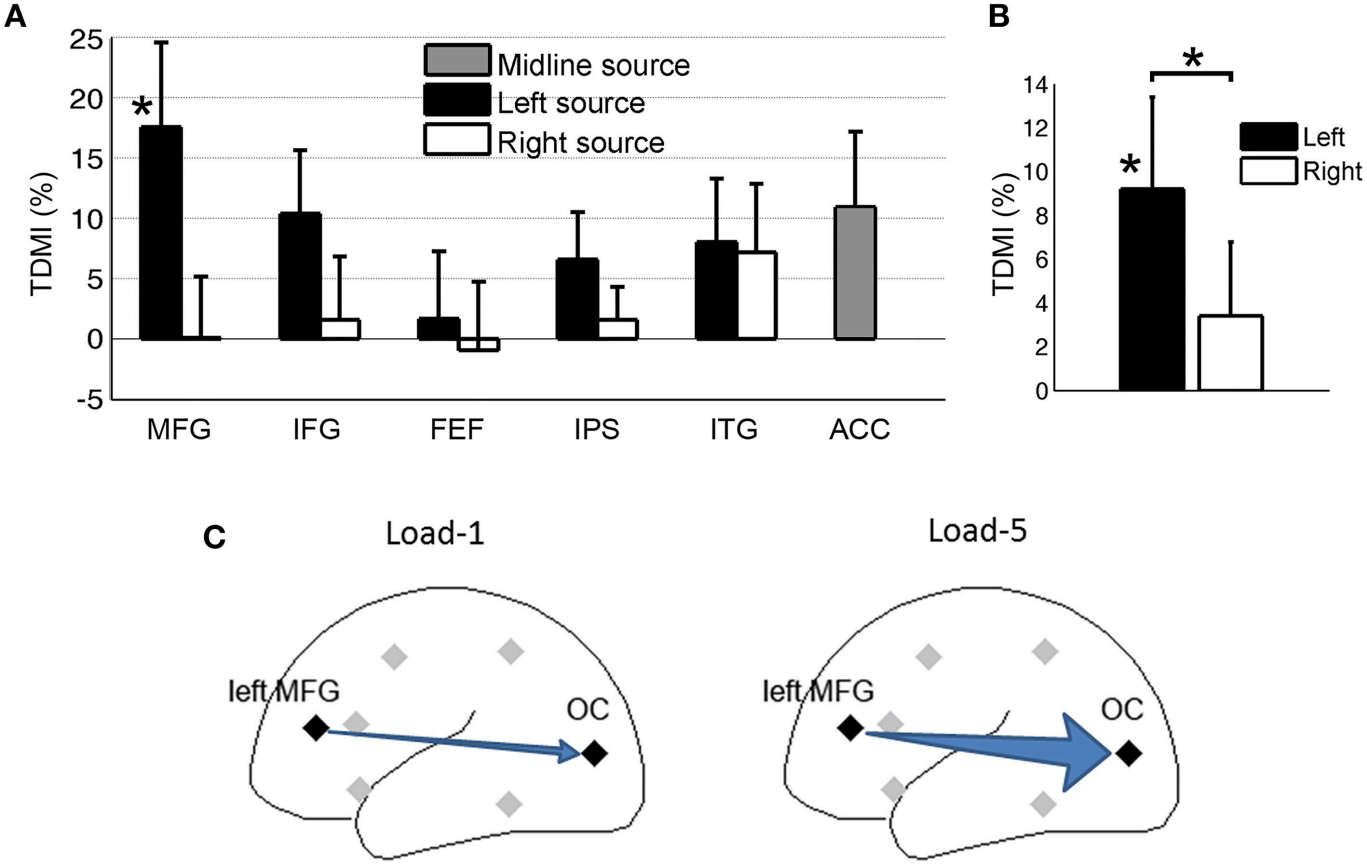
**Granger causality analysis at the source level for Experiment 2**. **(A)** Top-down modulation index (TDMI) of regional sources during working memory retention. **(B)** TDMI of left hemisphere sources and right hemisphere sources ^*^p < 0.05. **(C)** Schematic showing that alpha-band causal influence, left MFG→OC, significantly increased in load-5 condition compared to load-1 condition.

Figure 6A shows the TDMI in the alpha frequency range for each regional source. In general, the higher is the memory load, the stronger is the top-down causal influence. Statistically, the causal influences from the left MFG were significantly modulated by memory load (*p* = 0.031). Consistent with our finding at the scalp CSD level, the causal influence, left MFG→OC, was most strongly modulated by memory load, suggesting that the left MFG is likely the major source exerting top-down control of visual cortical activity during retention of working memory.

Averaging the top-down modulation indexes by hemisphere (Figure 6B), we found that the mean index from the left hemisphere sources was significantly larger than zero (*p* = 0.032), whereas the mean index from the right hemisphere sources was not significantly different from zero (*p* > 0.05). In addition, the left hemisphere mean index was significantly larger than the right hemisphere mean index (*p* = 0.034). This is again in contrast with Experiment 1 (Figure 4), where we found that, during anticipatory spatial attention, the right hemisphere sources exert more controlling influences on visual cortical activity. A schematic illustrating the result is shown in Figure 6C.

## Discussion

Goal-oriented modulations of visual alpha oscillations have been observed in numerous attention paradigms. According to the prevailing model, these modulations are effected by top-down influences from higher-order control areas in the brain. In this study we addressed three unanswered questions with respect to this hypothesis. What are the signals that mediate the top-down control? Are these top-down signals issued in a task-specific manner or by a common set of brain areas? What is the likely mechanism underlying the biasing actions of these signals? High-density EEG was recorded from subjects performing two experiments: (1) trial-by-trial cued visual spatial attention and (2) modified Sternberg working memory. Applying Granger causality, we found that (1) in both experiments, alpha oscillations mediate top-down influences, (2) in covert visual spatial attention, regions modulating visual alpha are lateralized to the right frontal cortex, with the dipoles located at right FEF and right IFG being the main sources of top-down signals, (3) in working memory retention, regions modulating visual alpha are lateralized to the left prefrontal cortex, with the dipoles located at the left MFC being the main source of top-down signals, and (4) these top-down signals may achieve the biasing effects via an inhibition-disinhibition mechanism. Below we discuss these findings in the context of the extant literature.

### Visual spatial attention

The dorsal attention network, including FEF and IPS (Kastner et al., 1999; Hopfinger et al., 2000; Corbetta and Shulman, 2002), is known to initiate and maintain the attentional set in visual spatial attention. The role of these cortical areas in sensory modulation has been studied with stimulation techniques. In monkeys (Moore and Armstrong, 2003; Armstrong et al., 2006), electrical micro-stimulation of the right FEF during passive viewing was shown to induce increased firing rates of visual cortical neurons. In humans, by combining fMRI with transcranial magnetic stimulation (TMS), it was found that stimulating the right FEF affected the blood oxygen level-dependent (BOLD) signals in visual cortex (Ruff et al., 2006). Furthermore, following the delivery of repetitive TMS to the FEF, spatial attention-related anticipatory visual alpha desynchronization was disrupted (Capotosto et al., 2009; Sauseng et al., 2011; Marshall et al., 2015), and this disruption led to changes in event-related potentials (ERPs) (Taylor et al., 2007).

The stimulation approach, while powerful, involves perturbing normal brain activity. Given that each brain area is connected with a vast number of other areas, disrupting one area's activity may lead to functional reorganization in the connected areas, making a focal interpretation of stimulation effects difficult (Robertson et al., 2003; Sack et al., 2005; Andoh and Martinot, 2008). In addition, assessing the causal role of multiple brain areas in a single stimulation study is not plausible. Multivariate analysis offers a nonperturbative alternative. Bressler et al. (2008) applied Granger causality measures to BOLD time series in a visual spatial attention task, and found that during attentional cueing, the activity of FEF and IPS predicts the activity of visual association regions. In the present study, applying Granger causality, we found that consistent with previous work, the regional source located at the right FEF was one of the major sources exerting top-down control over visual alpha activity in spatial attention, and this control is mediated by 10 Hz alpha oscillations. The right-hemisphere lateralization for the control of the visual cortex was supported by a TMS-fMRI study (Ruff et al., 2008), showing that TMS to the right FEF and right IPS affected processing in the visual cortex, but TMS to the left FEF and left IPS did not. It also agrees with lesion studies demonstrating that severe spatial hemineglect syndrome is common after right-hemisphere damage but rare after left-hemisphere damage (Mesulam, 1981; Heilman et al., 1985; Weintraub and Mesulam, 1987).

The lack of IPS→visual cortex modulation by attention is likely due to the limited spatial resolution of EEG. Although we projected the scalp EEG into the source space, each regional dipole source actually models a relatively large brain area. The true IPS effect could be attenuated by the activities of the adjacent brain regions that are unrelated to the present task. It is worth noting that recent studies using the simultaneous EEG-fMRI technique have implicated IPS in modulating visual alpha oscillation during attentional processing (Liu et al., 2016; Zumer et al., 2014).

### Working memory retention

Maintenance of verbal working memory requires that attention be directed internally and sensory processing be suppressed to reduce interference (Klimesch, 1999; Jensen et al., 2002; Thut et al., 2006; Tuladhar et al., 2007; Wyart and Tallon-Baudry, 2008). Although this suppression is also hypothesized to be implemented by top-down signals from higher order cortices (Gazzaley and Nobre, 2012; Ruff, 2013), no consensus has emerged regarding the sources of these signals. One possible source is lateral prefrontal cortex because past fMRI studies have shown that distraction suppression in working memory activates lateral prefrontal cortex (Jonides et al., 1998; Dolcos et al., 2007; McNab and Klingberg, 2008; Clapp et al., 2010). Additionally, disruption of left rather than right lateral prefrontal cortex led to reduced response accuracy in verbal working memory tasks (Mull and Seyal, 2001; Feredoes et al., 2006). Our result extends the previous findings by directly showing that the left MFG is the major source exerting top-down influence over visual cortex during retention of working memory. It further agrees with a recent transcranial direct current stimulation (tDCS) study showing that posterior alpha power can be modulated through stimulation over the left dorsolateral prefrontal cortex (DLPFC) during a two-back working memory task (Zaehle et al., 2011).

The left-hemisphere dominance in effecting verbal-working-memory-related-modulation of visual alpha activity coincides with the notion that left hemisphere is more involved in verbal information processing including verbal working memory (Smith et al., 1996; D'Esposito et al., 1998). Contrasting this finding with the right-hemisphere dominance in effecting spatial-attention-related-modulation of visual activity (Heilman and Van Den Abell, 1980; Weintraub and Mesulam, 1987), our finding suggests that top-down biasing signals over visual cortex are likely issued in a task-specific manner, rather than by the same set of frontal-parietal regions. Recently, a study (Falasca et al., 2015) using Granger causality analysis on MEG data revealed a similar pattern: when participants were judging coordinate spatial relations (a right-hemisphere dominant task), the top-down causal influence on the visual cortex were exerted only by the right frontal area; when participants were judging categorical spatial relations (a left-hemisphere dominant task), the right frontal area was not involved. Notably, even within working memory, the “top areas” are likely to be context-dependent. For spatial working memory previous neural imaging studies showed that the activation in the prefrontal cortex is predominantly right-lateralized (Smith et al., 1996; Reuter-Lorenz et al., 2000; Walter et al., 2003; Manoach et al., 2004). Zanto et al. (2011) showed that perturbing the right inferior frontal junction resulted in diminished top-down modulation of posterior activity and reduced accuracy in a visual working memory task.

### Long-range alpha band synchrony

Inter-regional interactions are thought to be mediated by long-range synchronized neural oscillations (Engel et al., 2001; Fries, 2005; Saalmann et al., 2012). Synchrony of alpha oscillations is functionally important (Palva and Palva, 2011; Mathewson et al., 2011; Jensen et al., 2014), as it is known to be modulated according to behavioral goals (Sauseng et al., 2005a; Freunberger et al., 2008; Doesburg et al., 2009; Buschman et al., 2012), and is correlated with task performance (Hummel and Gerloff, 2005; Bar et al., 2006; Freunberger et al., 2009; Zanto et al., 2011; Hamm et al., 2012). A recent simultaneous EEG-fMRI study further found that the long-range alpha synchrony was intrinsically linked to activity in the frontal-parietal control network (Sadaghiani et al., 2012). Our study contributes to this line of research by revealing that in both spatial attention and working memory the top-down causal influence from the frontal to the occipital cortex is mediated by long-range alpha synchrony whose function is to bias sensory neurons according to behavioral goals. Although in this study, the frontal sites issuing the top-down signals are shown to be different in the two different cognitive paradigms used here, given EEG's limited spatial resolution, whether the signals are transmitted via the cortico-cortical pathway or the cortico-thalamo-cortical pathway, as suggested by Saalmann et al. (2012), cannot be resolved.

### Possible mechanisms of sensory biasing

During working memory retention, visual cortex needs to be inhibited to gate out sensory input; the top-down Granger causal influence is increased in this case. Conversely, during covert visual spatial attention, visual cortex corresponding to the attended location is task-relevant and needs to be facilitated to more effectively process attended sensory input; the top-down Granger causal influence is decreased in this case. A parsimonious interpretation of this observation is that top-down Granger causality in the alpha band mainly reflects inhibitory influences. A reduction of alpha-band Granger causality with spatial attention represents a disinhibition of the visual cortex to increase excitability, whereas an increase of alpha-band Granger causality with working memory represents a further inhibition of the visual cortex. This interpretation is supported by anatomical evidence showing that fibers from higher-order areas to occipital lobe can arise from pyramidal neurons and terminate on inhibitory interneurons (Jones et al., 2000; Gonchar and Burkhalter, 2003). In addition, low frequency rTMS (≈1 Hz), which is known to be inhibitory (Hilgetag et al., 2001; Brignani et al., 2008), has been shown to cause long-lasting increases in alpha-band power and cortico-cortical alpha-band coherence, which leads to decreases in evoked potentials (Strens et al., 2002; Brignani et al., 2008). There is also evidence from studies in patients with visual hallucinations. During eye-close resting state when visual hallucinations are more likely to occur, EEG coherence between frontal and occipital regions was observed to be decreased in patients compared to healthy controls (Abraham and Duffy, 2001), suggesting that a reduction of influence from the frontal regions causes disinhibition of visual cortex which in turn leads to visual hallucinating (Abraham and Duffy, 1996). The inhibition-disinhibition hypothesis was further supported by examining the Granger causality in the pre-cue fixation period (−500 to 0 ms with 0 ms denoting cue onset) as a measure of baseline activity for the two experiments. The top-down causal influence in alpha band from the main sources was found to be lower in anticipatory attention and higher in high load working memory retention relative to their respective pre-cue baseline levels (see Supplementary Figure 3).

### Limitations and other remarks

First, a major limitation of this study is that the statistical significance of the main Granger causality effects is relatively low and does not survive multiple comparison correction. Typically, measures of brain connectivity are more sensitive to noise than measures of brain activation. Despite our best effort at preprocessing the EEG data, noise contamination remains inevitable. In addition, top-down control of sensory biasing could be a mechanism with considerable inter-individual variability. Although the number of subjects used in this study is reasonably large, even larger numbers may be necessary to overcome such variability. The fact that our findings are highly interpretable within the extant literature, however, suggests that these findings were not due to random chance. They could serve to motivate further investigations into mechanisms of attentional control using novel analytical techniques such as Granger causality. Second, EEG lacks access to neural firing activity. Any inference of possible mechanisms of sensory biasing is necessarily circumstantial and speculative. Moreover, in monkey studies, other sensory biasing mechanisms have been found. For example, Moore and Armstrong (2003) demonstrated increased neuron firing in V4 after microstimulation of FEF and Gregoriou et al. (2009) showed attentional enhancement of long-range coupling in the gamma frequency band between FEF and V4. A fuller understanding of this important issue will likely come from a synergistic approach integrating all available experimental preparations and methods. Third, although preprocessing is important for removing noise and artifacts, past work has shown that it may also distort the estimation of directional measures (Florin et al., 2010). We took care to minimize such distortion by applying only those preprocessing procedures that have been widely validated (e.g., ICA and zero-phase filter). Fourth, the directional of Granger causality modulation coincides with the direction of alpha power change, raising the concern of a power confound (Muthukumaraswamy and Singh, 2011; Schoffelen et al., 2011). For the present study, however, this is unlikely to be the case. Alpha power change is limited to the occipital sources. There is no alpha power change in other sources. If alpha power change in occipital sources is the reason underlying the observed Granger causality modulation then we should observe such modulation in all non-occipital sources. This did not occur. Only selected sources showed Granger causality modulation. Importantly, the roles of these sources are in concordance with the extant literature, as the foregoing discussions demonstrate. Fifth, like cross correlation, coherence and other measures of statistical associations, Granger causality is independent of the amplitude of the signals analyzed (Ding et al., 2006; see Supplementary Figure 5 for an illustration). While there might be differences in the magnitude of the three signals associated with the three dipoles at each source location, this property ensures that these differences are not biasing Granger causality estimation, and allows us to treat the causal influences from the three dipoles on an equal footing.

### Conflict of interest statement

The authors declare that the research was conducted in the absence of any commercial or financial relationships that could be construed as a potential conflict of interest.
