# Supplementary material for: Top-Down Control of Visual Alpha Oscillations: Sources of Control Signals and Their Mechanisms of Action
Source: Front Hum Neurosci. 2016 Jan 20;10:15. doi: 10.3389/fnhum.2016.00015 (PMC4718979; doi:10.3389/fnhum.2016.00015)
Supplement: Supplementary file 1 [file Presentation1.pdf]

Supplementary Material

Top-down Control of Visual Alpha Oscillations: Sources of Control Signals and Their Mechanisms of Action

Chao Wang, Rajasimhan Rajagovindan, Sahng-Min Han, Mingzhou Ding*

*** Correspondence:** Dr. Mingzhou Ding: MDing@bme.ufl.edu

We address six issues in this supplementary material section. The first issue concerns how well the dipoles represent local neural activity. We used source sensitivity maps in BESA to track whether the sensitivity of the modeled sources was sufficiently restricted to local current changes. To calculate source sensitivity, the software models unit brain activity at different locations throughout the brain, with source sensitivity defined as the fraction of power that is mapped on the selected source (Scherg et al., 2002). The test results are shown in Supplementary Figure 1. It can be seen that each regional source mainly account for brain activity around its location. The source sensitivity map does not depend on frequency. It can reflect the modeling sensitivity for alpha activity as well as activities of other frequency bands.

The second issue concerns the determination of the model order for the autoregressive models of the data. To choose a proper model order one usually seeks the minimum in the plots of Akaike information criterion (AIC) and Bayesian information criterion (BIC) versus model order. For electrophysiological data these two criteria often don't yield model orders in the physiologically meaningful range. In this paper we determine the proper model order by comparing the spectral estimates obtained by the MVAR model and that by the Fourier based method. Further this was done for data pooled across all subjects. Supplementary Figure 2A shows the power spectral density (PSD) for the bilateral occipital sources estimated by an MVAR model of model order 20 and by the FFT method. The two power spectra are

closely matched, indicating that the MVAR model provided a good representation of the stochastic processes behind the data. Supplementary Figure 2B shows the mean squared difference between the MVAR spectrum and the FFT spectrum as a function of model order. At the order of 20 and 21, we observed a minimum, indicating that at this model order, the match between the spectra estimated by two different methods is the closest. We therefore selected 20 as the model order for analysis.

The third issue concerns the addition of the pre-cue fixation period, defined to be (-500 ms 0 ms) with 0 ms denoting the onset of cue in both experiments, as a baseline condition. Supplementary Figure 3 shows the results for the main top-down influences. In spatial attention, we observed that the alpha-band causal influence right FEF→OC is significantly decreased in the ignore condition with further decrease in the attend condition compared to fixation ($p < 0.05$). This suggests that there is a weaker disinhibition for the ignore condition relative to baseline and a stronger disinhibition for the attend condition relative to baseline. This finding is consistent with the observation that alpha power is decreased for the ignore condition relative to baseline but is more strongly decreased for the attend condition. In working memory, we observed that the alpha-band causal influence, left MFG→OC, increased in the load-5 condition compared to fixation ($p = 0.06$), whereas the Granger causality values for fixation and for load-1 are similar ($p = 0.54$). This is also reasonable in the sense that load-1 is a very easy condition and does not require much additional top-down influence, whereas for the more difficult condition of load-5, stronger top-down control is required. Overall, by including the baseline condition, our findings remain consistent with the inhibition-disinhibition hypothesis.

The fourth issue concerns the topographic maps of alpha modulation and Granger causality modulation in terms of t-values. Supplementary Figure 4A and 4B are similar to Figures 3A and 3C and supplementary Figure 4C and 4D are similar to Figures 5A and 5C. This indicates that amplitude change and t-value yield similar information.

The fifth issue concerns a property of Granger causality, namely, it is independent of signal amplitude. A simulation approach was adopted to illustrate this. Consider the following autoregressive model:

$$X_t = 0.8X_{t-1} - 0.5X_{t-2} + 0.4Y_{t-1} + \epsilon_t$$

$$Y_t = 0.7Y_{t-1} - 0.75Y_{t-2} + \varepsilon_t$$

where ϵ_t and ε_t are two independent Gaussian white noise with zero mean and standard deviation of 0.3 and 0.2, respectively. The causal influence is by construction from Y to X. A data set of 100 realizations of 250 points each was obtained from the model. The sampling rate was 250 Hz. See Supplementary Figure 5 (left column) for sample time series and Y→X Granger causality spectrum. Next, we kept the time series X_t unchanged, multiplied Y_t by a factor of 3 to generate a new time series Z_t, and reanalyzed the data. As shown in Supplementary Figure 5 (middle column), although the magnitude of Z_t is 3 times that of the magnitude of Y_t, the Z→X and Y→X spectra are identical. In Supplementary Figure 5 (right column), we kept the time series Y_t unchanged, multiplied X_t by a factor of 3 to generate a new time series U_t. The Y→U and Y→X spectra are also identical.

The sixth issue concerns the location of the occipital dipoles. The coordinates of the occipital dipoles are based on previous fMRI studies of visual spatial attention (Hopfinger et al., 2000; LaBar et al., 1999). For Experiment 2, because visual activation is often not reported in past fMRI studies of verbal working memory, the same occipital dipoles were applied. We verified that the EEG alpha activity localized to these dipoles were modulated by working memory load (Supplementary Figure 6).

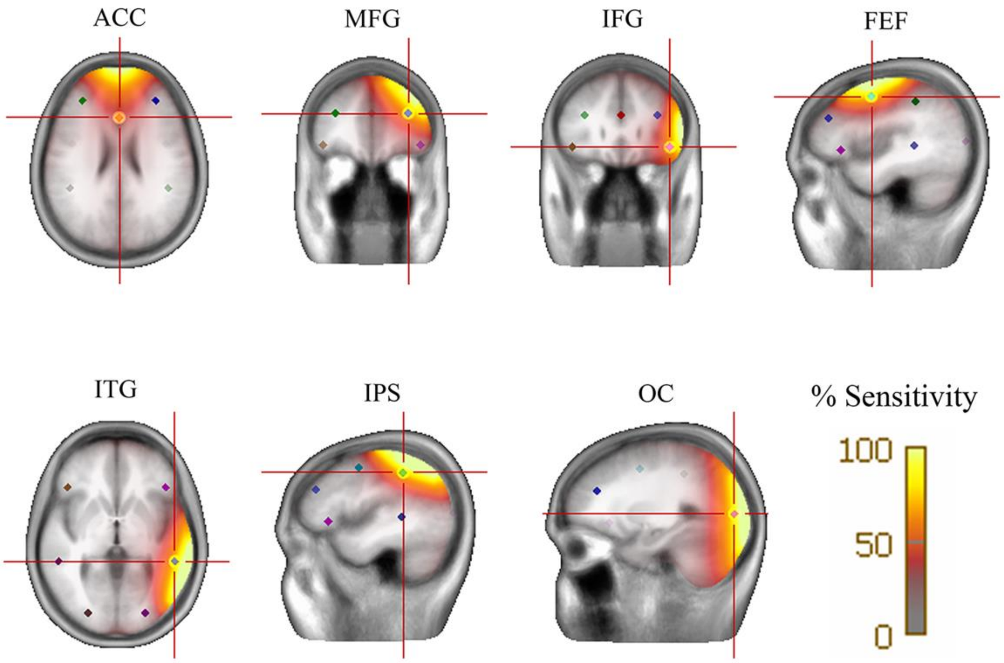

Supplementary Figure 1. Source sensitivity maps for the 13 discrete regional dipole sources used to model the data.

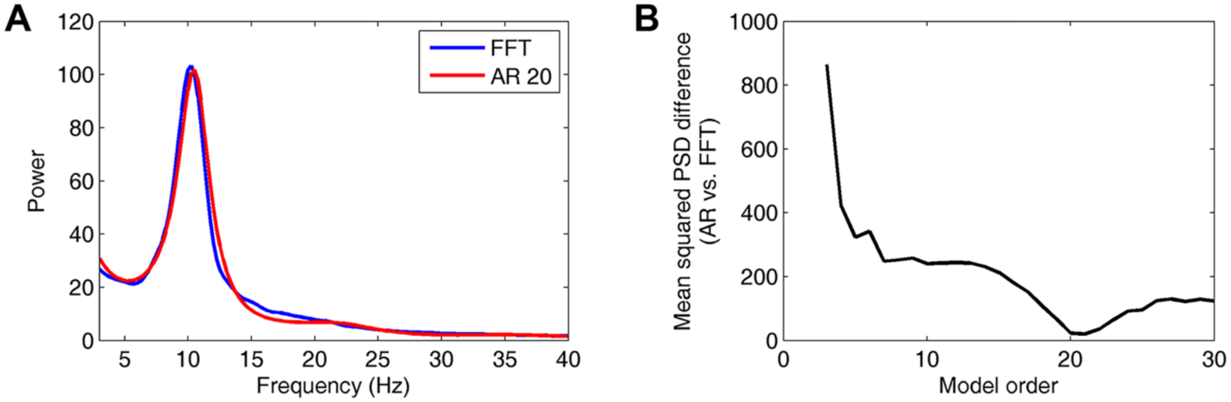

Supplementary Figure 2. (A) The power spectra estimated by an MVAR model of model order 20 and by the FFT method. (B) The mean squared difference between the MVAR spectrum and the FFT spectrum as a function of model order.

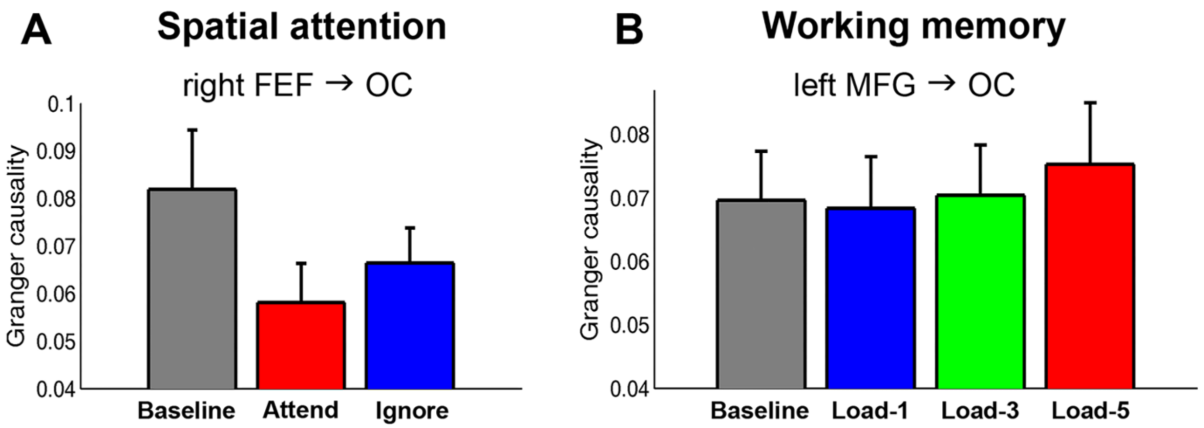

Supplementary Figure 3. (A) The right FEF→OC Granger causality in alpha band for baseline and different attention conditions during anticipatory attention (attend and ignore) in the spatial attention task. (B) The left MFG→OC Granger causality in alpha band for baseline and different load conditions during memory retention in the working memory task.

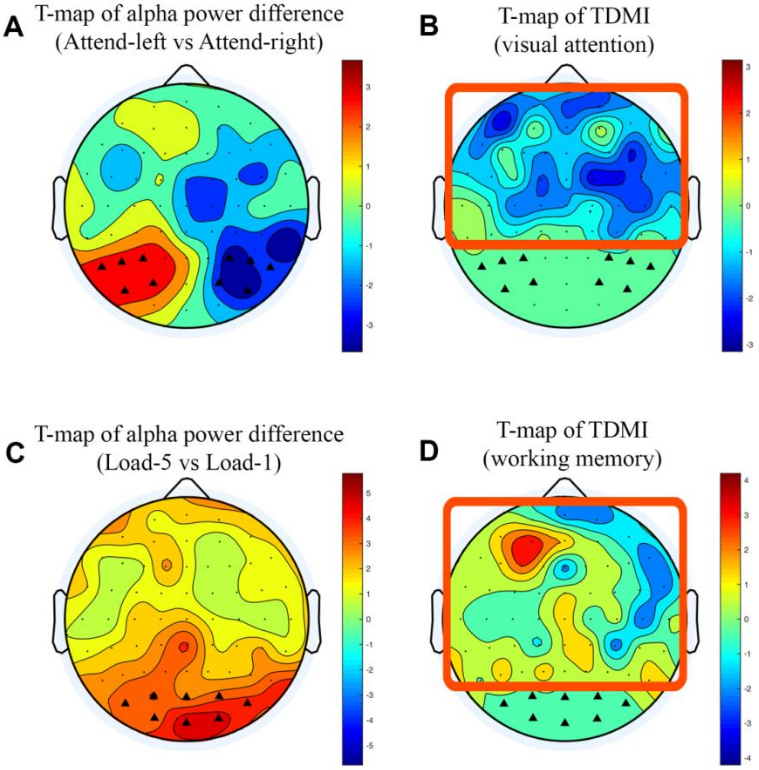

Supplementary Figure 4. Topographic maps of alpha modulation and Granger causality modulation in terms of t-values (t-maps). (A) T-map of alpha power difference by contrasting attend-left condition

against attend-right condition in Experiment 1. (B) T-map for top-down modulation index (TDMI) in the alpha band in Experiment 1. (C) T-map of alpha power contrasting load-5 condition against load-1 condition in Experiment 2. (D) T-map for top-down modulation index (TDMI) in the alpha band in Experiment 2.

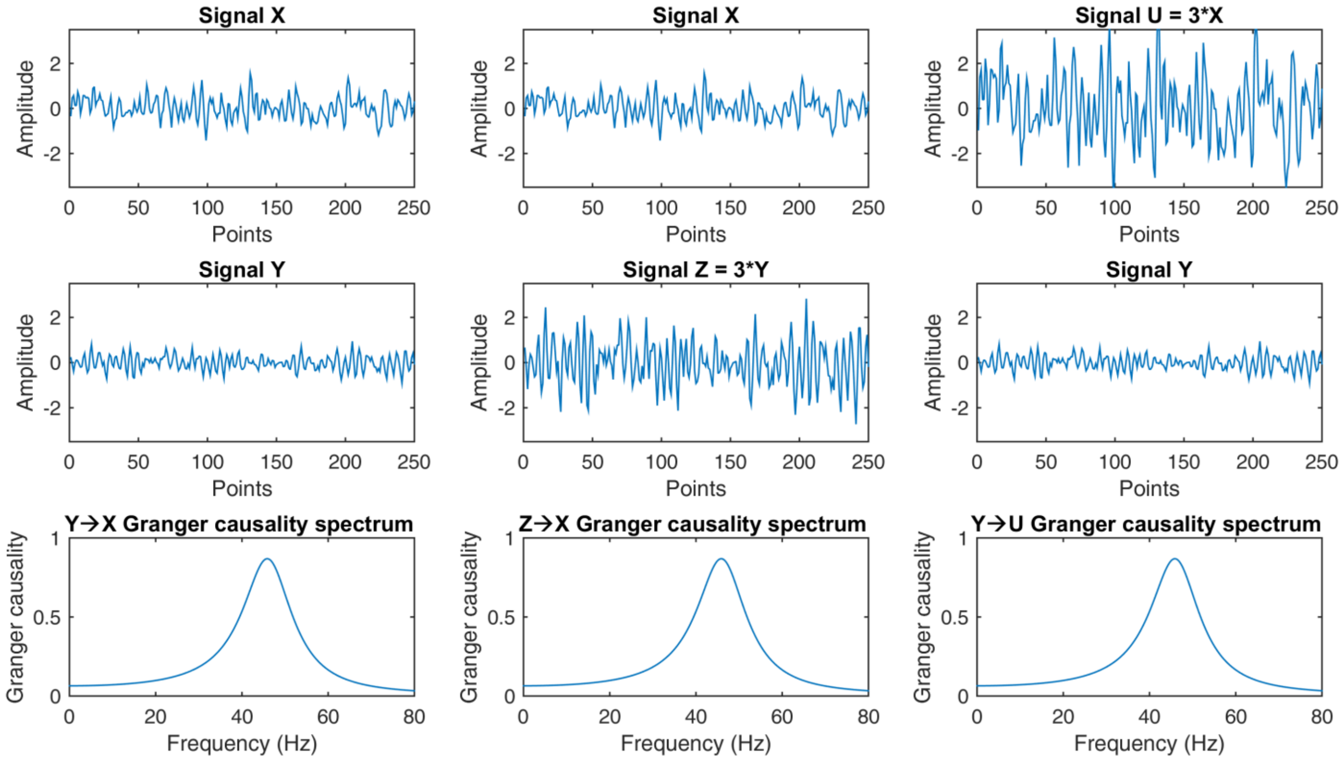

Supplementary Figure 5. Simulation results showing that Granger causality does not depend on the signal magnitude.

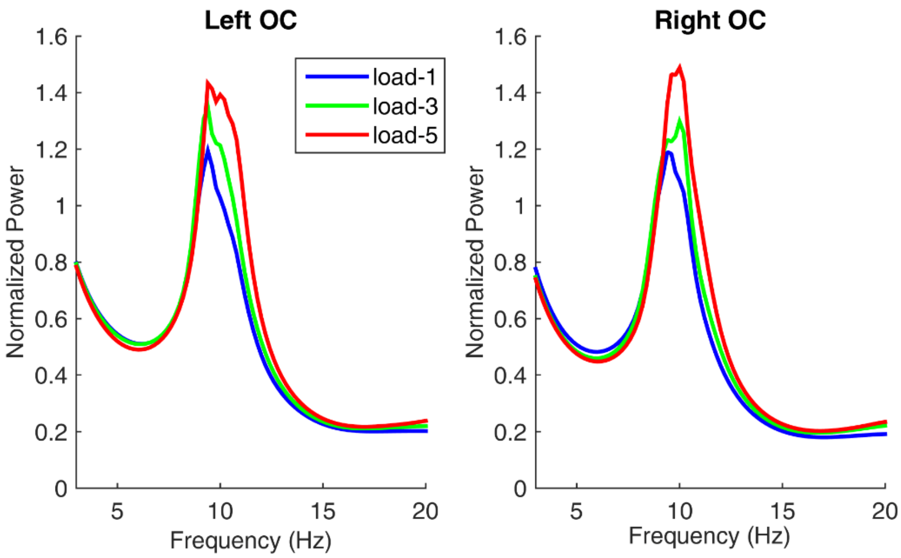

Supplementary Figure 6. Power spectra for the left and right occipital sources under load-1, load-3, and load-5 conditions in Experiment 2.
